# Fertilizing Nature: A Tragedy of Excess in the Commons

**DOI:** 10.1371/journal.pbio.1001124

**Published:** 2011-08-16

**Authors:** Allen G. Good, Perrin H. Beatty

**Affiliations:** Department of Biological Sciences, University of Alberta, Edmonton, Alberta, Canada

## Abstract

Globally, we are applying excessive nitrogen (N) fertilizers to our agricultural crops, which ultimately causes nitrogen pollution to our ecosphere. The atmosphere is polluted by N_2_O and NO_x_ gases that directly and indirectly increase atmospheric warming and climate change. Nitrogen is also leached from agricultural lands as the water-soluble form NO_3_^−^, which increases nutrient overload in rivers, lakes, and oceans, causing “dead zones”, reducing property values and the diversity of aquatic life, and damaging our drinking water and aquatic-associated industries such as fishing and tourism. Why do some countries show reductions in fertilizer use while others show increasing use? What N fertilizer application reductions could occur, without compromising crop yields? And what are the economic and environmental benefits of using directed nutrient management strategies?

## Abstract 2

Why has nitrogen fertilizer use declined in some countries while increasing in others, despite significant environmental harm? Proper crop management strategies offer environmental and economic benefits without sacrificing yields.

In his 1968 seminal paper, “The Tragedy of the Commons,” the late Garrett Hardin argued that individuals, acting in rational pursuit of their own self-interest, will sacrifice the long-term viability of a shared resource for short-term gain. “Ruin is the destination toward which all men rush, each pursuing his own best interest in a society that believes in the freedom of the commons.” In the case of pollution, he wrote, “Here it is not a question of taking something out of the commons, but of putting something in – sewage, or chemical wastes into water” [1]. Perhaps one of the best examples of this “over-contribution” is nitrogen (N) fertilizers, where individual rational behaviour (i.e., applying high fertilizer rates to maximize short-term economic yield) can cause long-range harm to the environment. The true cost of applying high rates of N fertilizers in order to maximize overall yield is already apparent in the form of global climate change. The incentive to over-apply N fertilizers is likely to continue, as both the Food and Agriculture Organization (FAO) and the United Nations (UN) have predicted high future demand for cereal production, especially within the developing nations, due to predicted increases in populations and dietary shifts.

In developed countries, crop yields have nearly reached their biological maximum and increasing fertilizer use is unlikely to provide any significant additional gains. In contrast, in developing countries, there is still a large yield gap. Although we need to increase crop yields to feed the growing global population, we also need to do this in an environmentally sustainable way. We cannot increase our yields by increasing N fertilizer application (not even in areas of the world that still have an exploitable yield gap) at the expense of the ozone layer or marine life. Certainly, while regions with an N balance surplus can reduce N fertilizer application rates without yield losses (i.e., Denmark), other regions will need to increase their N use (i.e., sub-Saharan Africa), but still use best management practices. N balanced countries may also be able to reduce N fertilizer rates without yield loss by employing new technologies such as improved plant varieties, region-specific farming practices, time-release N fertilizer, drip irrigation, crop rotation, bioinoculants, and similar approaches.

## Nitrogen Is a Key Aquatic and Atmospheric Pollutant

Nitrogen is the key limiting nutrient for most crops and many aquatic and terrestrial ecosystems. Unfortunately, the massive increase in anthropogenic N introduced into the environment, largely via N fertilizers, has had significant negative environmental consequences [2],[3]. The link between agriculture and nitrate pollution is well established with impacts on drinking water [4],[5] and the eutrophication of fresh water and marine ecosystems, including the proliferation of harmful algal blooms and “dead zones” in coastal marine ecosystems [6]. For example, in the United States, 89% of total N inputs into the Mississippi River come from agricultural runoff and drainage [6]. In addition, agriculture plays a substantial role in the balance of the three most significant anthropogenic greenhouse gases (GHGs): carbon dioxide (CO_2_), nitrous oxide (N_2_O), and methane (CH_4_). The global warming potential (GWP) of these gases can be expressed in CO_2_ equivalents. The GWPs of N_2_O and CH_4_ are 296 and 23 times greater, respectively, than a unit of CO_2_ [7]. Of these, N_2_O is the most important gas emitted by fertilizer use, because of its large CO_2_ equivalent influence on GWP. In the US, agriculture contributed 68% of the country's N_2_O emissions in 2009, but only 3.6% of the total US GHG emissions [8].

Rather than try to fix the consequences of N fertilizer overuse, a better solution would be to employ better management strategies, such as tillage type, rate and timing of N fertilizer application, better sources of N fertilizer such as timed-release N fertilizer, bioinoculants or biological N fixation, and more N-efficient crop plants. Although reducing or eliminating anthropomorphic N pollution will necessitate a multi-dimensional approach, we will focus mainly on one approach, N fertilizer application reduction.

## The Value and Consumption of N Fertilizers Are Both Rising

The global value of N fertilizers has increased from US$32B annually in 1987 to over US$80B annually, and even conservative estimates project it to increase to US$150B by 2030 (Table 1). Overall global consumption has increased 18% over the past 20 years, due in most countries to an increase in cereal production [9]. The N balance within countries and regions reflects the N input to output ratio as surplus, neutral, or deficit. A surplus N balance leads to NH_3_, N_2_O, NO_3_^−^, and or NO pollution while a deficit N balance leads to low soil fertility from depleting soil nutrient pools, resulting in poor crop yield [10]. While overall some countries, such as the US, have become fairly balanced in their N input to output, with little increase in overall N fertilizer consumption since 1975, there are still major areas of cropland that are rated as having high N balances, resulting in soils highly susceptible to losses of N_2_O to the atmosphere [11]. Other regions, such as the European Union (EU), have had significant fertilizer N consumption reductions [12]. The large reduction in N fertilizer consumption was achievable in these countries because of an initial N balance surplus that polluted the water quality to an unacceptable level up to 1987 ([12], Table 1). The EU successfully implemented nutrient reduction programs by developing best nutrient management practices (BNMPs; [13])—such as coordinating fertilizer requirements and application methods and rates to particular crops, soils, and soil water status—that have improved the quality of ground water, rivers, and lakes [14]. China is an example of a region with high N balance surpluses and an N fertilizer consumption rate that is still on the rise. There are other regions in the world that display N balance deficits, for instance the countries of sub-Saharan Africa, which have chronically nutrient-poor soils and under-use N fertilizers [3].

**Table 1:** The N fertilizer costs and consumption of specific countries for past use and future forecasts.

World				EU		Denmark		China	
Year	Price($ Mton^-1^)	Total Consumption(MMt N)	Value(US$B)	Total Consumption(MMt N)	Consumption(kg ha^−1^)	TotalConsumption(MMt N)	Consumption(kg ha^−1^)	TotalConsumption(MMt N)	Consumption(kg ha^−1^)
1987	425	75.8	$32.2	30.3	127	0.367	142	18.6	138
1997	610	81.3	$49.6	15.5	101	0.238	120	25.4	185
2007	795	100.6	$80.0	13.3	114	0.172	75	34.8	247
*2012*	*869*	*103.2*	*$89.7*	*13.0*	*114*	*0.170*	*74*	*37.6*	*267*
*2020* a	*980*	*110.7*	*$108.5*	*13.0*	*114*	*0.170*	*74*	*42.4*	*302*
*2030* b	*1220*	*126.9*	*$154.8*	*13.0*	*114*	*0.170*	*74*	*54.5*	*388*
*2050* c	*1500*	*151.6*	*$227.4*	*13.0*	*114*	*0.170*	*74*	*69.0*	*487*

Italicized text represents future estimates and are based on no increase in consumption (EU and Denmark) or a linear increase in consumption, equivalent to the rate of increase between 1987 and 2007 (world and China) (http://www.fao.org/corp/statistics/; accessed 16 June 2010).

a Estimates of World N consumption in 2020 are 110 MMt [55], 112 MMt [56], and 135 MMt [57].

b Estimates of World N consumption in 2030 are 125 MMt [56].

c Estimates of World N consumption in 2050 are 135 MMt [58] and 236 MMt [57].

MMt, million metric tons.

Given the tremendous costs associated with N fertilizer over-application, it's helpful to consider why the EU has managed an overall reduction in N fertilizer while countries such as China have increased their use, how N fertilizer usage could be reduced without compromising crop yields, and what the economic and environmental benefits of directed nutrient management strategies might be.

## Why Has There Been an Overall Decrease in N Fertilizer Application in Some Countries and Not in Others?

Within the EU, there has been a 56% decrease in total fertilizer use between 1987 and 2007, including a significant decrease in N application per hectare (Table 1). In Denmark, for example, producers have decreased the applied nitrogen by 52% since 1985, resulting in a 47% reduction in ammonia emissions [14]. How was this achieved? Danish agriculture was forced to employ sustainable agricultural methods after the adoption of the Nitrate Directive in 1987 [15],[16], which mandated the use of BNMPs to reduce nitrate levels in drinking water. After evaluating the most appropriate BNMPs for specific crops, soils, and different cropping systems and using nutrient budgeting models (including organic N sources), Danish regulatory bodies identified improved agronomic practices (such as restricting fall N fertilizer applications, which are often leached as toxic emissions over winter). From this research, the government enacted legislation outlining specific N inputs and management practices for each crop [14]. EU producers are now required to provide detailed N farm budgets before they can receive Common Agricultural Policy (CAP) subsidy payments [17].

Recent reports on Chinese agricultural methods, in contrast, indicate that N fertilizer use is much higher than required for optimal yield, in some cases up to 600 kg N ha^−1^ [18]. The government encouraged producers to use more fertilizer to attain higher yields and support China's domestic food security. However, recent estimates of fertilizer usage in China suggest that a reduction of 30% to 50% in applied fertilizer would not necessarily reduce yields [18]. Assuming a conservative reduction of 10% below current usage, by 2020 China could reduce its fertilizer use by 11.5 MMt per year, compared to the predicted increase in N application. This would result in savings to Chinese producers of US$11.3B annually (Table 2).

**Table 2:** Total N consumption, economic, and environmental costs for the US, China, India, and the world.

Year	Actual/Predicted Consumption(MMt N)	Value(US$B)	Proposed Reduction (from 2007)	Reduced Consumption (MMt N)	Excess N Applied (MMt N)	Value (US$B)	CO_2_ GWP (MMt)^a^	CO_2_ GWP Value(US$B)	Environmental Cost(US$B)
**World**									
1987	75.8	32.2							
2007	100.6	80.0							
2020	110.7	108.5	10%	90.5	20.2	19.8	94	1.4	8.7
2030	126.9	154.8	20%	80.5	46.4	56.6	215.8	3.2	24.9
2050	151.6	227.4	20%	80.5	71.1	106.7	330.7	5.0	46.9
**US**									
1987	9.5	4.1							
2007	14.5	11.5							
2020	16.7	16.3	5%	13.8	2.9	2.7	13.5	0.2	1.2
2030	19.9	24.3	10%	13.0	6.9	8.4	32.1	0.5	3.7
2050	23.5	35.2	10%	13.0	10.4	15.7	48.4	0.7	6.9
**China**									
1987	18.6	7.1							
2007	34.8	27.6							
2020	42.4	42.0	10%	31.3	11.5	11.3	53.5	0.8	5.0
2030	54.5	66.5	20%	27.8	26.7	32.5	124.2	1.9	14.3
2050	69.0	103.5	20%	27.8	41.2	61.7	191.6	2.9	27.1
**India**									
1987	5.7	2.4							
2007	14.4	11.5							
2020	19.1	18.7	10%	13.0	6.1	6.0	28.4	0.4	2.6
2030	24.7	30.2	20%	11.5	13.2	16.1	61.4	0.9	7.1
2050	31.4	47.1	20%	11.5	19.9	29.8	92.6	1.4	13.1

Economic costs are calculated based on the current and predicted cost of N fertilizer. Environmental costs are equal to 44% of the value of the excess N applied.

a The GWP of N_2_O based on a 1% of excess applied N being lost as N_2_O-N; excess N applied MMt N x 0.01×(44/28) x GWP of N_2_O (296). CO_2_ GWP Value (1 Tonne CO_2_  =  US$15). Price of N fertilizer (1987 = US$425; 2007 = US$795; 2020 = US$980; 2030 = US$1,220; 2050 =  US$1,500).

## Reducing N Fertilizer Application without Reducing Yield

In the US, the United Kingdom, and other countries, rice (*Oryza sativa*), maize (*Zea mays*), wheat (*Triticum aestivum*), and barley (*Hordeum vulgare*) have been grown experimentally to determine their N response to increasing fertilizer applications (commonly expressed as an N response curve; Table 3). These long-term studies demonstrate that implementing BNMPs can allow for a reduction in N fertilizer application with no loss to yield, even in N balanced systems. Also, for those developing countries that need to increase their N fertilization rates, there is still a requirement to implement local specific management strategies to increase yield and reduce future excessive application rates (Table 3).

**Table 3:** Improvements in nitrogen use efficiency in crop plants during field trials.

Crop	Year	N Fertilizer Rate(kg N ha^−1^)		% Decr. in N Fertilizer	% Incr. in Yield	PFP_N_ a		% Incr. PFP_N_ a	N Management	Ref.
		High	Low			From High Rate	From Low Rate			
**Americas**										
Maize	2000–03	191		0	60	55	72	31	BMP versus Illinois state averages	[49]
Maize	1980–00	145		0	35	42	57	36	Improved BMP and use of modern hybrids	[36]
Wheat	1994–96	250	180	28	21	20	34	67	Farmers’ practice versus BNMP in Northern Mexico	[59]
Barley	2007	169	107	37	NC^b^	46	71	54	Field trials in Alberta, Canada	[46]
Maize	1998–99	250	187	25	NC^b^	43	57	33	Improving N management Kansas & Nebraska, US	[60]
Maize	2001–03	134	101	25	NC^b^	50	67	34	Michigan, N_2_O emissions double after using more N than the low rate	[37]
Maize	2007–08	180	135	25	NC^b^	44	59	25	Michigan, using the lower N rate reduces N_2_O emission by 44%	[38]
Maize	2005–07	150	90	40	−8^c^	69	105	34	Eastern Canada, N_2_O emission at low N rate were half that of high N rate	[41]
**Europe**										
Wheat	2003–07	200	174	13	1	27	30	11	High rate versus optimal N rate at the N:grain price ratio = 5	[42]
Cereals	1985–02	na		na	na	36	44	22	Average UK cereal NUE declining use of N fertilizers	[61]
Wheat	Prior to 2001	200	160	20	NC^b^	52	64	24	Using balanced nutrients, “Law of Minimum”	[13]
**Asia**										
Rice	1987–99	108	76	30	NC^b^	45	66	47	Fertilizer rate reduced, NUE variety in Japan	[62]
Rice	1998–99	167	133	20	8	37	49	32	FFP versus SSNM^d^ at 21 farms in China	[63]
Rice	1997–99	117	112	4	7	49	52	6	FFP versus SSNM^d^ at 179 sites in Asia	[33]
Rice	2003–06	300	200	33	3	27	41	52	East China farmers' N practice versus opt N fertilization	[18]
Rice	1995–98	70	53	24	NC^b^	30	40	33.	On-farm studies, increasing plant population density	[32]
Wheat	2003–06	325	128	61	5	18	47	161	North China Plain farmers' N practice versus opt N fertilization	[18]
Maize	2003–06	263	158	40	5	32	56	75	North China Plain farmers' N practice versus optimum N fertilization	[18]

a PFP_N_, kg grain per kg kg N applied.

b NC, no change in yield.

c Slight decrease in yield at the low N fertilizer rate versus high N fertilizer rate.

d FFP, farmers' fertilizer practice; SSNM, site-specific nutrient management.

Many field studies have been done in various regions of the world, analyzing the optimum BNMPs for the specific region, including fertilizer rate, for a variety of crops. All of these studies indicate that reductions in fertilizer usage, in those situations where it is being applied in excess, can occur without any loss in yield (see Box 1).

**Box 1. Reducing Fertilizer Applications**

**Rice**: For China, it was suggested that a reduction in N fertilizer application of 30% to 60% could be implemented for wheat, maize, and rice while still maintaining current crop yields [18]. The authors argued that reductions in N fertilizer usage would cause no significant reduction in yields in the rice/wheat and wheat/maize double-cropping systems in eastern and northern China, respectively. This is because the current N fertilizer application rates are upwards of 600 kg N ha^-1^ and much of this N is lost from the crop-soil system by leaching into the aquatic environment and atmospheric emissions ([18] and references within).

Japanese rice farmers use less N fertilizer currently on their crops than in the past, with no loss in yield. In the early 1990s, a fall in rice prices induced rice farmers to decrease their N fertilizer application rates from 109 kg N ha^-1^ in 1985 to 80 kg N ha^-1^in 1997, while still maintaining rice yields. This success was attributed to the reduction of excessive fertilizer application and the use of an N use efficient rice variety called Koshihikari, which maintains high yield under a lower N regime [30]. Currently in Japan, nitrogen use efficiency (NUE) of rice has increased over 30% from 1985 to recent years [31].

On a broader scale, it has been demonstrated [32] that there was no correlation between countries that had high levels of yields for rice and the NUE of that country. For example, Japan had high rice yields and high NUE, whereas China had high yields but low NUE. In a multinational field trial program (179 farm sites in seven countries) for intensive rice production organized by the International Rice Research Institute (IRRI), rice grain yield was increased by 7% by balanced fertilizer use, although less N was applied [33].

**Maize**: There have been many N fertilization studies conducted with maize in the US, and some selected examples are shared here (Table 3). One study found that both the currently recommended N application rate (168 kg ha^-1^) and the farmers' use (197 kg ha^-1^) exceeded the profit maximizing level of N by a minimum of 35% [34]. Minnesota farmers were able to reduce nitrogen use in corn by 21% without any reduction in crop yield [35]. Based on US Department of Agriculture statistics for US maize yield and fertilizer N used for corn production, from 1980 to 2000 US maize yields increased by 35% without significant increases in N fertilization levels [36]. A three-year Michigan corn study using different fertilizers, different fertilizer management strategies, and nine N fertilizer application rates (from 0 to 292 kg N ha^-1^) showed that using 101 kg N ha^-1^ maximized grain yield while minimizing N_2_O emissions, whereas using 134 kg N ha^-1^ or more increased N_2_O emissions significantly [37]. These authors concluded that N_2_O emissions could be reduced, without a yield penalty, by reducing N fertilizer inputs to a level that just satisfies the crops N requirement. A study conducted from 2007 to 2008 in Michigan at multiple commercial corn farms examining N_2_O response to six different N fertilizer rates (0-225 kg N ha^-1^) showed that high rates of N fertilization led to (on average) nonlinear increasing rates of N_2_O loss without economic yield gains [38]. When old versus modern maize hybrids were examined, modern hybrids had an optimal N application rate that was 18% less than older hybrids (160 kg ha^-1^ versus 195 kg ha^-1^), despite the fact that the modern varieties also had significant improvements in yield, in the range of 20% [39]. The research and education/extension programs of many of the land grant universities have been effective at reducing excess applications of N fertilizers; however, even *The Economist* was quick to point out that “Western countries have complacently cut back on the work done in universities and international institutions. It was a huge mistake. Basic farm research helps the whole world—and is a bargain” [40]. However, there have been studies to suggest that farmers applying BNMPs or new fertilizer technologies can reduce their N fertilizer application with no loss in yield [23],[38]. A seven site-year study conducted on corn farms in Ontario, Canada, determined the effects of N fertilizer rate and timing on yield and N_2_O emissions [41]. The authors determined that although there was a slight increase in yield when fertilizer rate increased from 90 to 150 kg N ha^-1^, cumulative N_2_O emissions also doubled.

**Wheat**: There have been a number of studies that have demonstrated that modern wheat varieties have improved NUE (Table 3). Modern UK wheat varieties have shown a 14.6% to 18% increase in NUE, depending on the N conditions [42], while modern Spanish wheat varieties had a 24% to 29% increase in NUE (as measured by PFP_N_; [43]). A number of other UK wheat varieties have been evaluated and significant differences were determined in total N uptake and grain N uptake efficiency, depending on the N application rate [44]. These differences in NUE were primarily determined by greater yield, not increased concentrations of N in the plant material.

**Barley**: A number of studies have demonstrated that modern barley varieties have improved NUE (Table 3). Modern UK barley varieties, under optimally applied N conditions, had a 27% increase in NUE [41]. Also, modern Argentinean barley varieties had a 24% to 29% increase in NUE (as measured by PFP_N_) over older varieties [45]. Eight years of data for different varieties of spring barley grown in Canada were analyzed and the best performing varieties had a 7% to 17% improvement in NUE over the mean for all varieties [46].

## Economic and Environmental Benefits of Using Directed Nutrient Management Strategies

The economical optimal N rate (EONR) is the rate of fertilizer that allows for the maximum economic yield [19],[20]. After the fertilizer price has been included, a lower N fertilization rate than the maximum yield rate should be applied. What is now needed is a way to measure the environmental and economic optimal N rate (EEONR). This N rate takes into account the N fertilizer price plus the cost of the N lost to the environment. The environmentally optimal N application rate for maize was recently calculated, suggesting that a rate of 25 kg ha^−1^ less than the economic optimal N application rate would reduce GHG emissions [21]. The Iowa State University Agronomy Extension in 2004 recommended another approach, the maximum return to N (MRTN), using a range of economical N inputs for US Midwest corn farmers that take into account both N fertilizer prices and corn prices [11]. Although this approach does not directly take into account environmental costs, it does suggest a range of fertilizer rates, on average, 185 kg N ha^−1^ (the high profitable N rate) to 158 kg N ha^−1^ (the low profitable N rate) that are both below the well-used and recommended yield goal N rate of 250 kg N ha^−1^, or more [11]. This reduction in N fertilizer rate also reduced N pollution of the ecosystem. Many studies conducted in the US, especially through the corn-belt region, show that loss of N to crops can be reduced by reduced N fertilizer application, management practices, and type of fertilizer used [22],[23]. Nutrient management strategies take into consideration not only N fertilizer application rate, but also factors including type of tillage, type of N fertilizer, and rotation with N fixing crops. N fertilizer is needed to maintain or increase crop yields; however, depending on the tillage system and crop rotation used, a high N application rate can decrease farmer profits and increase N_2_O emissions [23]. For example, corn farmers in Colorado using a conventional tillage and continuous corn (CT-CC) management system can reduce both GWP and increase net profits by reducing N fertilizer application. If those same farmers switched to a no-till corn-bean rotation system, they could further reduce GWP and increase profits but at a higher N fertilizer rate than for CT-CC [23].

The type of N fertilizer applied can directly affect N_2_O emissions as well. Research conducted in Colorado for two years on N_2_O emission rates from irrigated no-till-corn grown with enhanced-efficiency N fertilizers versus conventional dry urea and liquid urea-ammonium nitrate showed that the enhanced-efficiency N fertilizers reduced N_2_O-N emissions while maintaining yield [24]. Yields of Minnesota potatoes were maintained while reducing N_2_O emission by using single, pre-plant applications of polymer-coated urea for N fertilizer compared to multiple split applications of conventional uncoated urea [25]. As well as maintaining yields with fewer N_2_O emissions, the N fertilizer costs were reduced due to the need for only a single application versus multiple applications with conventional urea.

N fertilizer (organic and inorganic) that is not taken up by crop plants can be lost to the environment through nitrification/denitrification of ammonium/nitrate (respectively) by soil microbes. N runoff and leaching of nitrate into waterways (aquifers, rivers, lakes, and oceans) and ammonia volatization into the atmosphere can also occur. While we recognize that losses vary dramatically, depending on multiple variables, we made a number of simple assumptions to model these N losses from excess N fertilizer applications and calculate their economic costs to the environment. For most cereal crops, only 30% to 50% of applied N is actually taken up by the plant [26],[27]. Therefore, we assumed that plants take up approximately 40% of the available N with the remaining 60% as surplus N. The fate of the surplus N can include becoming an environmental pollutant (Table 4), or held in soil as organic or inorganic N, depending on the soil and N type.

**Table 4:** N losses to the environment and the calculated economic value of these costs for the US.

N Component	Average (%)	Range (%)	Environmental Costs (US $B)
Plant product	40	30–50	NA
Tier 1 N_2_O-N emission factor	1	0.003–0.03	1.01B^a^
Leaching and runoff (Nitrate)	20	15–28	3.6B
N_2_O from volatized ammonia	20	15–25	0.47B^b^
Lost or denitrified	19	15–83	ND
Total	100	—	5.1B (44%)

Total N applied in the US in 2007 was 14.5 MMt at a value of US$11.5B.

a 14.5MMt x 0.01×(28+16 g mol^−1^ / 28 g mol^−1^ ) × 296×$15/t  =  US$1.01B.

b (14.5 MMt N fertilizer US) / (83 MMt N fertilizer globally) × (0.6 Tg N_2_O formed from ammonia volatilization)  = 0.11 Tg N_2_O volatized ammonia in the US per year. 0.11 MMt N_2_O×296×US$15/t =  US$0.47B.

We attempted to determine the environmental and associated economic costs of N applications, using the US as an example (see Box 2). The choice of the US was based on the fact that there are better data available. Using both fertilizer use and price projections, we evaluated the cost savings associated with reducing N budgets such that they matched the appropriate regional fertilizer recommendations (Table 2). All countries analyzed in Table 2 were assigned a neutral, or reduction in N fertilizer use (5% to 20%), based on analysis of their overuse of fertilizers in the selected literature we have cited (Table 3). While this analysis included only four major regions/countries, these collectively account for 74% of global fertilizer use [9]. Based on this analysis, savings of US$19.8B per year and US$56B per year are attainable by 2020 and 2030 respectively, assuming no change in the area of farmed land.

**Box 2. The Environmental Cost of Excess N Applications**

Global atmospheric N_2_O concentrations have increased from the pre-industrial level of 270 ppb to 319 ppb in 2005, with agriculture (fertilizer use and animal production) as the primary source of this added N_2_O. N_2_O can remain in the atmosphere for approximately 114 years [47]. The FAO has predicted that by 2030 global N_2_O emissions from fertilizer and manure application will increase by 35% to 60% [38]. For the loss of N by emission of N_2_O via denitrification, we used the Intergovernmental Panel on Climate Change [47] linear Tier 1 N_2_O default emission factor of a 1% loss of applied N as N_2_O-N (1 kg of N_2_O-N emitted per 100 kg of applied N) which takes into consideration N_2_O-N emissions from N applied as mineral and organic fertilizers, crop residues and N mineralized from soil due to loss of soil carbon [47]. It should be noted, however, that N_2_O emissions can vary due to not only N fertilizer rate, but also soil type (texture, drainage, pH), soil organic carbon levels, climate, type of N fertilizer applied, method of fertilizer placement, and crop type grown [47],[48]. Several studies conducted in the US and Canada have shown that N_2_O-N emission rates can be nonlinear, especially at higher N fertilizer rates, showing that higher N fertilizer rates can produce exponential N_2_O emissions [11],[38],[41]. Since this 1% N_2_O-N emission factor is an estimate, it may under-represent the actual N_2_O emission rate when the N fertilizer rate exceeds the crop or soil uptake ability [22]. Globally in 2005, N fertilizer use was approximately 93 MMt and caused an estimated emission of 1.46 MMt of N_2_O, equal to 433 MMt of carbon dioxide equivalents (CO_2_e) [48]. In 2007, in the US, 14.5 MMt of N were applied to crops [9], representing 0.228 MMt of N_2_O emissions, having the GWP of 67.4 MMt of CO_2_e. Therefore, the partial environmental cost of soil N_2_O emissions can be estimated based on the CO_2_ equivalency. Carbon dioxide credits are traded as commodities on the European and New Zealand CO_2_ exchanges, so they have a monetary value. When the value for CO_2_ is taken as US$15/ton, the N_2_O emissions in the US equates to a value of US$1.01B annually. Although N is also lost as NO_2_ and N_2_ (20% of applied N may be lost as N_2;_ [49]) via nitrification and denitrification, there is no directly measurable cost associated with these types of N loss, so we did not include these in our partial estimates of environmental costs. As well, N_2_ does not have a negative environmental impact on the ecosystem.

One measure to determine the economic cost of excess nitrate from runoff and leaching would be to look at the economic and social impact excess N has against specific industries. As an example, about 8% of the N applied in the US corn-belt is being directly exported into the Gulf of Mexico via the Mississippi River [50]. This lost N has both a direct economic cost to the agricultural producers, but also has an indirect negative impact on other economic activities. In the Gulf of Mexico, commercial and recreational fisheries currently generate US$2.8B annually. However, one half of the shellfish and many oyster beds have either been permanently closed or declared indefinitely off-limits by health officials as a result of N pollution [51]. Therefore, we estimated the cost to the Gulf marine economy to be US$1.4B annually. An analysis of the economic cost of eutrophication of US freshwaters as it pertains to loss of recreational activities, property value, threatened and endangered species recovery efforts, and drinking water was recently completed [52]. Dodds et al. [52] provide a conservative estimate of the eutrophication cost to be US$2.2B annually for the US fresh waterways. Therefore, in total, a conservative cost estimate for excess runoff and leaching in the US is US$3.6B.

Losses of ammonia from N fertilizer application can be as high as 50% to 80%, depending on climate, type of fertilizer used, application method, and soil type [53]. Livestock manures and urea fertilizers tend to volatize the most ammonia globally at 23% and 21%, respectively [53]. Ammonia is not considered to have a direct GWP, so a direct cost of ammonia volatization is difficult to calculate. However, ammonia emissions affect air, water, and land quality, and can lead to “acid rain,” which causes marine and soil ecosystems to become acidic and in turn contributes to aquatic eutrophication and soil acidification [49]. High levels of ammonia and ammonium can reduce plant diversity, increase plant predation by insects, and cause serious human diseases, including cardiovascular and lung diseases and asthma [53]. Ammonia has a short life span in the atmosphere and is either dry deposited locally to the site of emission or converted in the atmosphere to ammonium (NH_4_^+^), nitric oxides (NO_x_), and N_2_O. Ammonium can accumulate in clouds and be wet deposited in regions distant from the site of emission. Globally, synthetic fertilizers and agricultural crops account for 12% of total ammonia emissions [54]. Of the 83 MMt of N fertilizer used globally in 1996, an estimated 0.6 Tg of N_2_O was formed from atmospheric ammonia oxidation. Assuming similar losses in the US, 0.11 Tg of N_2_O was formed in 2007, with an indirect GWP cost of US$0.47B.

We determined the environmental costs from excess N to conservatively be 44% of the cost of the total N applied in the US. We then used this value in Table 2 to model the environmental costs associated with excess applied N for the world, the US, China, and India. While these gross cost estimates may not be accurate for any one crop, they provide a starting point for discussion. We fully recognize the challenges of accurately estimating site-specific N losses. However, the important goal is to identify the costs associated with the various types of N pollutants. These cost estimates can then be used to develop economic tools to ensure that the environmental costs are integrated into BNMPs.

## Several Simple Proposals to Reduce N fertilizer Use

It is clear from many studies that when N application rates are in balance, N losses via N_2_O emissions and leached nitrate are reduced to a minimum, depending on the cropping system [17],[28]. Although dry land cereal production in Canada is usually based on a single, pre-planting application of N fertilizer and mobilization of the applied N is by rain-fed moisture, many cropping systems allow revised application rates, which, along with more careful monitoring of the 4Rs (right source, right time, right place, right rate), can result in significant reductions of N losses that harm the environment. Clearly, by using BNMPs, the producer benefits from reduced costs while everyone benefits from an improved environment.

In order to successfully optimize the use of N fertilizers (both agronomically and environmentally), we propose several simple approaches. First, fertilizer use requirements need to be reassessed in virtually all agricultural systems, from an economic and environmental perspective. Second, economic and environmental models need to be integrated and be made user-friendly, particularly in those developed and developing countries where excessive N use occurs. Third, countries need to ensure that government programs do not discriminate against producers who voluntarily choose to use less fertilizer. For example, crop insurance often requires the farmer to apply fertilizers at the recommended (but potentially out-dated) rate, otherwise they will not be compensated for potential crop losses. Fourth, we need to find economic tools to better inform and drive changes in N application rates. It is easy to say that reducing rates will help reduce N_2_O emissions, but the producer does not benefit economically from that, unless there is some form of payment for reducing N applications. This is effectively providing a global ecological service. Some countries, such as Austria and Finland [29], have begun to implement “green taxes” (i.e., taxes on fertilizers and agrichemicals). However, at a minimum, we need to eliminate “negative green incentives”, which often provide direct subsidies to farmers to use fertilizers. Regardless of the tools used to promote change (legislative, economic), education programs need to be put in place immediately to promote the environmental and economic benefits of the optimal use of N fertilizer.

## In Conclusion

Through a combination of the 4R BNMPs and advances in fertilizer technology and plant genetics, it may be possible to reduce global N application rates by 20% by 2050, saving US$150B annually, compared to business as usual. Unlike many of the challenges faced by agriculture, reducing excess nutrient applications (as demonstrated by the EU) is within our ability. Finally, farmers, scientists, and economists need to communicate more efficiently to promote the use of the EEONR and BNMPs while providing scientific data and leadership to address this issue.

## Abbreviations

BNMP: best nutrient management practices
CAP: common agricultural policy
CT-CC: conventional tillage and continuous corn
EEONR: environmental and economical optimal N rate
EONR: economical optimal N rate
EU: European Union
FAO: Food and Agriculture Organization
GHG: greenhouse gas
GWP: global warming potential
MRTN: maximum return to N
N: nitrogen
NUE: nitrogen use efficiency
UN: United Nations
4R: right source, right time, right place, right rate
